# Feasibility of Hepatitis C Elimination in China: From Epidemiology, Natural History, and Intervention Perspectives

**DOI:** 10.3389/fmicb.2022.884598

**Published:** 2022-06-02

**Authors:** Zeyu Zhao, Meijie Chu, Yichao Guo, Shiting Yang, Guzainuer Abudurusuli, Roger Frutos, Tianmu Chen

**Affiliations:** ^1^State Key Laboratory of Molecular Vaccinology and Molecular Diagnostics, School of Public Health, Xiamen University, Xiamen, China; ^2^CIRAD, Intertryp, Montpellier, France

**Keywords:** hepatitis C, epidemiology, natural history, interventions, elimination

## Abstract

Hepatitis C imposes a heavy burden on many countries, including China, where the number of reported cases and the incidence of hepatitis C virus (HCV) increased yearly from 2005 to 2012, with a stable trend after 2012. The geographical distribution of HCV infections varies widely in China, with the northwest and southwest regions and the Henan Province showing a high disease burden. Elderly, men, sexually active people, drug users, migrants, blood transfusion recipients, and renal dialysis patients have become the target populations for hepatitis C prevention and control. It is important to improve the diagnosis rate in high-risk groups and asymptomatic people. Identifying secondary HCV infections, especially in HCV patients co-infected with the human immunodeficiency virus (HIV) is a priority of hepatitis C prevention and control. Enhancing universal access to direct antiviral agents (DAAs) treatment regimens is an effective way to improve the cure rate of HCV infection. For China to contribute to the WHO 2030 global HCV elimination plan, strategic surveillance, management, and treatment program for HCV are needed.

## Introduction

Hepatitis C is a blood-borne disease caused by the hepatitis C virus (HCV), leading to acute or chronic infection. Newly infected individuals are usually asymptomatic. Approximately 30% (15%–45%) of people with acute infection clear the virus by themselves within 6 months, with the remaining 70% (55%–85%) developing chronic hepatitis C (World Health Organization, 2020). HCV infections have led to a serious disease burden. According to the World Health Organization (WHO), around 71 million people worldwide are infected with chronic HCV, and there were 1.75 million new cases of HCV infection worldwide in 2015 (World Health Organization, 2017a). The WHO proposed a hepatitis C elimination plan in 2016 that projects a 90% reduction in new HCV infections and a 65% reduction in deaths globally by 2030 (World Health Organization, 2020). Many countries around the world are working to achieve this goal.

China is actively responding to WHO’s plan through the Healthy China Initiative (2019–2030), aiming to increase blood-borne disease testing, high-risk occupational screening, and universal safe sex (The State Council of the People’s Republic of China, 2019). However, China still has one of the highest rates of hepatitis C infection in the world (Hajarizadeh et al., 2013; World Health Organization, 2017a). HCV infections showed a slow upward tendency from 2012 to 2017 (Gao et al., 2019). In addition, 9,795,000 HCV infections and 45,300 HCV deaths have been reported in 2016, which were both high prevalence and high mortality rates (World Health Organization, 2017a; CDA Foundation, 2020).

There is no vaccine against HCV and prevention measures rely mostly on screening improvement and health promotion (Gomaa et al., 2017). However, routine medical examinations in China do not include HCV detection, resulting in a very low number of HCV cases detected through active surveillance. Only 18% of people with HCV were diagnosed in China in 2016 (CDA Foundation, 2020). The main measure against HCV is medical therapy. Direct antiviral agents (DAAs) have changed the traditional treatment regimen for HCV infection and have been shown to reduce the disease burden of HCV, thus offering hope for a successful HCV elimination.

Despite their effectiveness, DAAs have not been well implemented in China due to several factors. Therefore, it is critical to further explore effective measures to reduce HCV morbidity and mortality in China and thus contribute to the WHO 2030 HCV Elimination Plan. Previous studies have explored the proportion of asymptomatic and symptomatic individuals with acute and chronic HCV infection. Further studies should elucidate the course of infection in patients to clarify the natural history of HCV infection and to distinguish between acute and chronic stages of HCV infection. This distinction, which can be effective delineation of important populations and segments, can be useful for the elimination of hepatitis C.

## Factors Influencing the Feasibility of Hepatitis C Elimination in China

Understanding the epidemiological characteristics of HCV infections, such as population, temporal, and spatial distribution, can help to take targeted prevention and control measures against HCV. In recent years, the incidence of hepatitis C in China has been slowly increasing. In addition, the unbalanced spatial distribution of disease burden and genotypes further increases the challenge of preventing and treating HCV infection. Although the detection of HCV has improved in recent years, the reporting rates in China have not improved significantly (European Association for the Study of the Liver, 2018). A large number (85%–90%) of people with HCV are currently asymptomatic (Ozaras and Tahan, 2009). This phenomenon has led to the majority of HCV-infected patients ignoring them that have hepatitis C, and the detection rate of active HCV is very low. This greatly increases the difficulty of preventing and controlling hepatitis C. Therefore, there is a need to further investigate key areas and populations and propose targeted strategies.

## Epidemiological Characteristics of Hepatitis C in the Mainland of China

### Temporal Distribution

**Figure 1:**
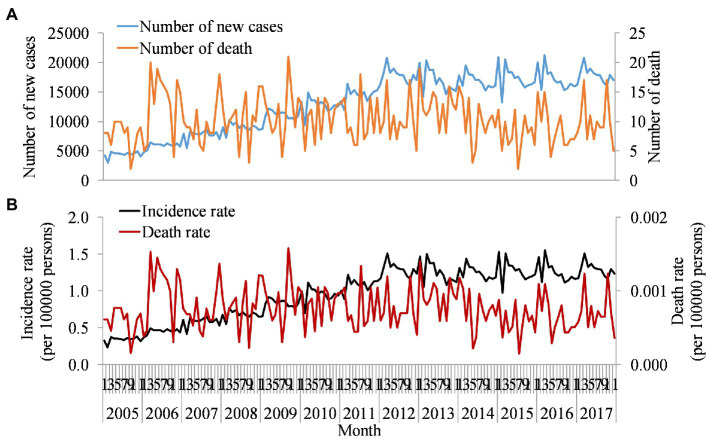
Monthly reported number of new cases, deaths, incidence, and death rates of hepatitis C in China, 2005–2017. Orange line in plot **(A)** used left axis represents new cases, and blue line used left axis represents death cases of hepatitis C virus (HCV) per month reported by Chinese Centre for Disease Control and Prevention (CDC). Plot **(B)** is the rate calculated by new cases and death cases in plot **(A)** after adjusting for population.

Previous studies found that the number of reported hepatitis C cases in China increased slowly from 1997 to 2003 with an annual increase of 27.89% and rapidly increased from 2004 to 2011 with an average annual increase of 48.79% (Qin et al., 2013). According to the Chinese Center for Disease Control and Prevention, a study found that there was a sharp increase from 52,927 cases in 2005 to 201,622 cases in 2012, with a steady trend from 2012 to 2017 (Figure 1A). Previous studies also found consistent with our results (Liu et al., 2018). Furthermore, the number of new cases showed seasonality, with a peak in March every year. The trend in incidence rate was also similar (Figure 1B). The number of deaths due to hepatitis C also showed fluctuations with an average of 10 deaths reported per month. According to an epidemiological survey of serum specimen testing in China in 2006, the prevalence of HCV infection in the total population was 0.43%. However, the actual reporting rate for that year was only 5.41 per 100,000 (Chen et al., 2011). Only one in 10 infected individuals may be detected.

### Spatial Distribution

**Figure 2:**
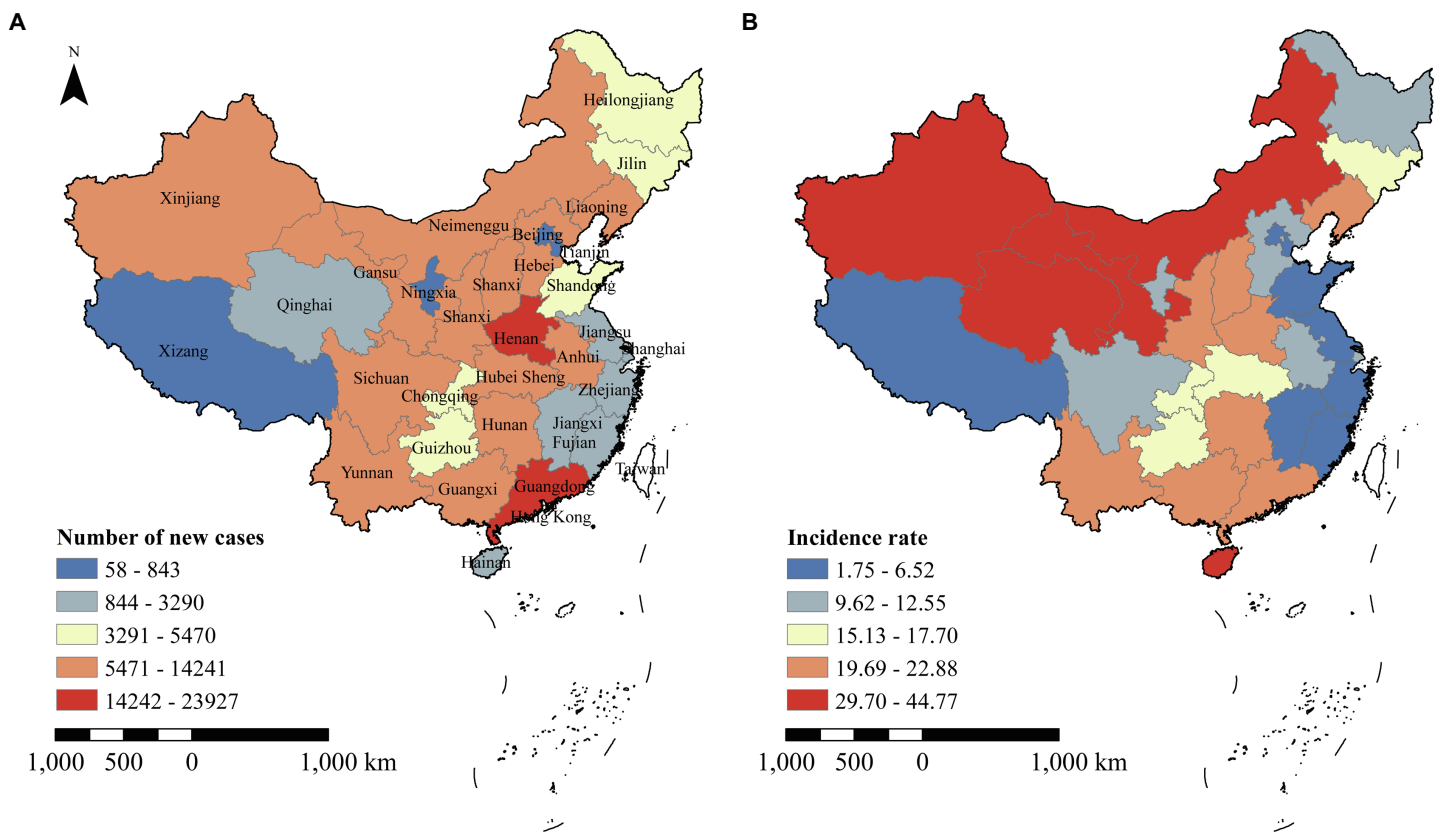
Number of new cases and incidence of HCV infection reported in China in 2017. Plot **(A)** represents the HCV cases reported by China CDC in 2017. Plot **(B)** represents incidence rate calculated by the population of each province in China.

The geographical distribution of hepatitis C in China varies widely. The top three provinces with the greatest number of new confirmed cases in 2017 were Guangdong (23,927 cases), Henan (21,446 cases), and Hunan Province (14,241 cases). The three provinces with the lowest new confirmed cases were Ningxia Hui Autonomous Region (803 cases), Tianjin City (784 cases), and Tibet Autonomous Region (58 cases; Figure 2A). The geospatial distribution of the incidence and new cases of HCV varies considerably among provinces due to differences in population size. The geographical distribution of incidence rates in China in 2017 showed a trend of high in the northern regions and low in the southern regions. The higher incidence rates concentrated in the northwestern regions of China (including Xinjiang Uyghur Autonomous Region, Qinghai, Inner Mongolia Autonomous Region, Ningxia Hui Autonomous Region, Gansu, and Shaanxi Province). The highest incidence of HCV infection was recorded in the Xinjiang Uyghur Autonomous Region (44.77 cases/100,000 people), while the lowest incidence rate was found in Beijing (3.87 cases/100,000 people; Figure 2B). A previous study also found that the highest incidence of hepatitis C was observed in Xinjiang (Moriyama and Rahman, 2018). Sociocultural and economic factors may play a key role in HCV transmission (Zhou and Zhou, 2013; Moriyama and Rahman, 2018); specifically, low socioeconomic conditions, poor public health programs, and indigenous customs contribute to an increased burden of HCV (Abdelhakam and Othman, 2018). A previous study found that healthcare access in ethnic minority regions was worse than in the non-minority region (Mali Xia et al., 2014; Wang and Pan, 2016). Some ethnic minorities, located in border areas, may have more drug trafficking and use, potentially contributing to the high incidence rate of HCV infection (Zhou et al., 2016). Besides, the governments in developed areas may invest more money and resources in healthcare and people’s health awareness than in underdeveloped areas (Zhou and Zhou, 2013). There may be urban–rural inequality of opportunity in healthcare in China (Ma et al., 2021). Therefore, the Chinese government should strengthen economically and culturally appropriate, regional-specific interventions to curb the HCV-transmitted infection epidemic, especially in the northwestern and southwestern regions as well as in the Henan Province.

In addition, the regional HCV infection rates among Chinese injecting drug users (IDUs) also varied considerably, with the lowest HCV infection rate among IDUs being 11.43% (Shaanxi) and the highest being 90.77% (Hubei). HCV prevalence is also high in IDUs in the Yunnan Province, Guangxi Zhuang Autonomous Region, Hunan Province, Xinjiang Uygur Autonomous Region, etc. (Xia et al., 2008). The lowest hepatitis C infection rate among non-injecting drug users is 0% (Anhui Province) and the highest is 40.00% (Fujian Province; Bao and Liu, 2009). Another study conducted HCV detection among 2,000 injecting drug users in Yunnan Province and found that 77% of the participants were infected with HCV (Zhou et al., 2012). Furthermore, the prevalence of HCV seropositivity among IDUs in China was the highest in the southwest (77.7%, 95%CI: 69.9%–85.4%), followed by the south (76.2%, 95%CI: 65.9%–86.4%). In terms of specific regions, the Sichuan Province had the highest prevalence of HCV antibodies (91.7%, 86.6%–95.3%), followed by the Guangxi Zhuang Autonomous Region (86.1%, 95%CI: 81.8%–90.4%; Bao et al., 2019).

### Population Distribution

**Figure 3:**
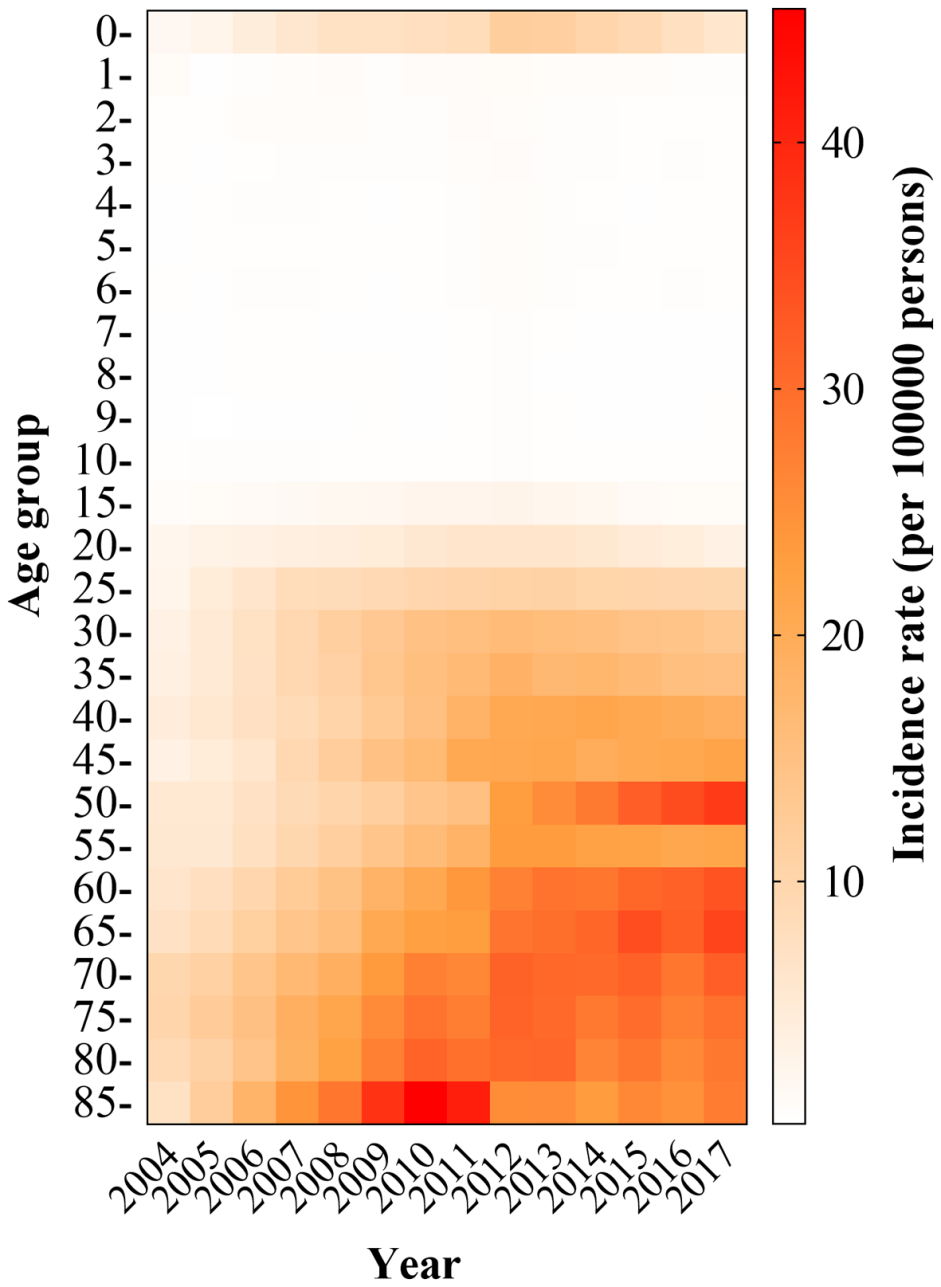
Incidence of reported HCV infection in different age groups in China, 2004–2017. The reported incidence rate by different age groups was obtained from a website (https://www.chinacdc.cn/).

The age distribution of reported HCV incidence varies. The main characteristic was that HCV incidence tends to affect younger people, while the prevalence remained high in older adults, especially in those aged 60. In 2004, the prevalence was higher in people aged 25–44 and 55 or older, while in 2017 the prevalence was more evenly distributed in all age classes above 25 (Figure 3). The incidence of HCV infection in infants (0–1-year-old) rose and then fell, with a peak in 2012 (11.71 cases/100,000) during the 2004–2017 period. Mother-to-infant transmission has become the most common routine of hepatitis C virus infection, with risk factors including titer of HCV RNA, IgM positivity, high viral load, active drug use, and HIV coinfection in the mother (Ohto et al., 1994; Dal Molin et al., 2002; Syriopoulou et al., 2005; Squires and Balistreri, 2017). There was a significant upward trend in prevalence between 2004 and 2012, with a smoothing trend in prevalence among people aged 85 years after 2012. The incidence of hepatitis C was a clear upward trend since 2012 among people aged 50–55, with the highest incidence (37.061 cases/100,000) in 2017. In addition, a previous study has found that the prevalence of hepatitis C generally increases with age (Li et al., 2020). Another study also demonstrated that the seropositivity was correlated with high age (≥60 years; Xu et al., 2019).

A study in the Liaoning Province showed that the seropositivity was significantly higher in men than in women, with detection rates ranging from 0.18 to 2.40% for men and 0.20 to 2.07% for women (Li et al., 2020). One study reported a prevalence ratio of 1.6:1 for men and women (Cornberg et al., 2011). This gender difference is directly related to risk behaviors, such as unprotected sex in men who have sex with men (MSM), sharing syringes, and tattooing (Rao et al., 2019).

Hepatitis C is present in various occupational groups in China, and retirees, farmers, workers, and accounted for the largest proportion (Xin-rong and Qing-long, 2019; Yang et al., 2019; Tian et al., 2020; Wang and Han-bing, 2020). The prevalence among female sex workers ranged from 0.32 to 1.14%, and it was even higher among female sex workers with lower socioeconomic status (Chen et al., 2015). The prevalence was 0.3%–0.5% for male truck drivers and passengers and 0.2% for pregnant women and young students (Wang et al., 2013).

The use of infected syringes is currently one of the main routes of HCV transmission in China. The prevalence was 66.97% among IDUs and 18.30% among non-injecting drug users (Bao and Liu, 2009). A study showed a high disease burden of HCV infection among IDUs in China, with high seropositivity of 71.6% (95%CI: 65.7%–77.6%; Bao et al., 2019). Another study found that there was a 60.1% (95% CI: 52.8%–67.0%) prevalence of hepatitis C among outpatients on methadone maintenance treatment (Zhuang et al., 2012). The prevalence of HCV infection among IDUs in China is higher than that in general drug users. The main reason is certainly the sharing of needles, syringes, or other drug-related paraphernalia, leading to cross-contamination (Midgard et al., 2016; Chinese Society of Hepatology, Chinese Medical Association, and Chinese Society of Infectious Diseases, Chinese Medical Association, 2020). This indicates that effective interventions for the prevention and control of HCV infection among IDUs should be implemented.

Human immunodeficiency virus and HCV have the same mode of transmission and share common risk factors. Immunocompromised individuals are vulnerable to HCV infection, and high active antiretroviral therapy (HAART) is associated with hepatotoxicity. Therefore, HIV/HCV co-infection is common. Previous studies have found a 24.7% HCV prevalence among HIV-infected individuals (Yu et al., 2020). As of October 2020, a cohort study of HIV patients in Guangxi Zhuang Autonomous Region, China, found that 8.1% co-infected with HCV (Jia et al., 2022). A cross-sectional survey in Yunnan Province reported 6.5% of a total of 5,922 HIV/AIDS cases were infected with HCV (Li et al., 2021). As of October 2021, the number of people living with HIV in China reached 1.14 million and is still increasing (Xian, 2021), implying that there might be 74,100–281,580 cases of HIV/HCV co-infection in China.

Blood transmission is another major route of HCV infection, with a prevalence of 166.56 per 100,000 among first-time blood donors and 15.21 per 100,000 among regular blood donors (Fu et al., 2019). The prevalence of hepatitis C in hemodialysis patients was approximately 10%, which is higher than the general population (Jadoul et al., 2019).

With the transformation of China’s economy and the proposal of the National New Urbanization Plan (2014–2020), the orderly growth of the agricultural transfer population has been encouraged. As a result, the scale of domestic migration in China has been constantly expanding. Rural workers began to migrate to urban areas for better employment opportunities. However, a large proportion of them are limited to 3D (i.e., dirty, dangerous, and difficult) occupations. Migrants remain a socially vulnerable group with high health risks. One study indicated that the prevalence of hepatitis C among Chinese internal migrants reached 0.45%, which is 3.8 times higher than in the general population (Zou et al., 2014).

Among other high-risk groups for HCV infection, MSM are represented with a higher prevalence of 0.7%–1.2% than the general population (Chow et al., 2014). HCV prevalence among clandestine sex workers ranged from 0.7 to 0.9% and 0.8 to 0.9% among men attending sexually transmitted disease (STD) clinics (Wang et al., 2013).

### Genotypic Distribution

**Figure 4:**
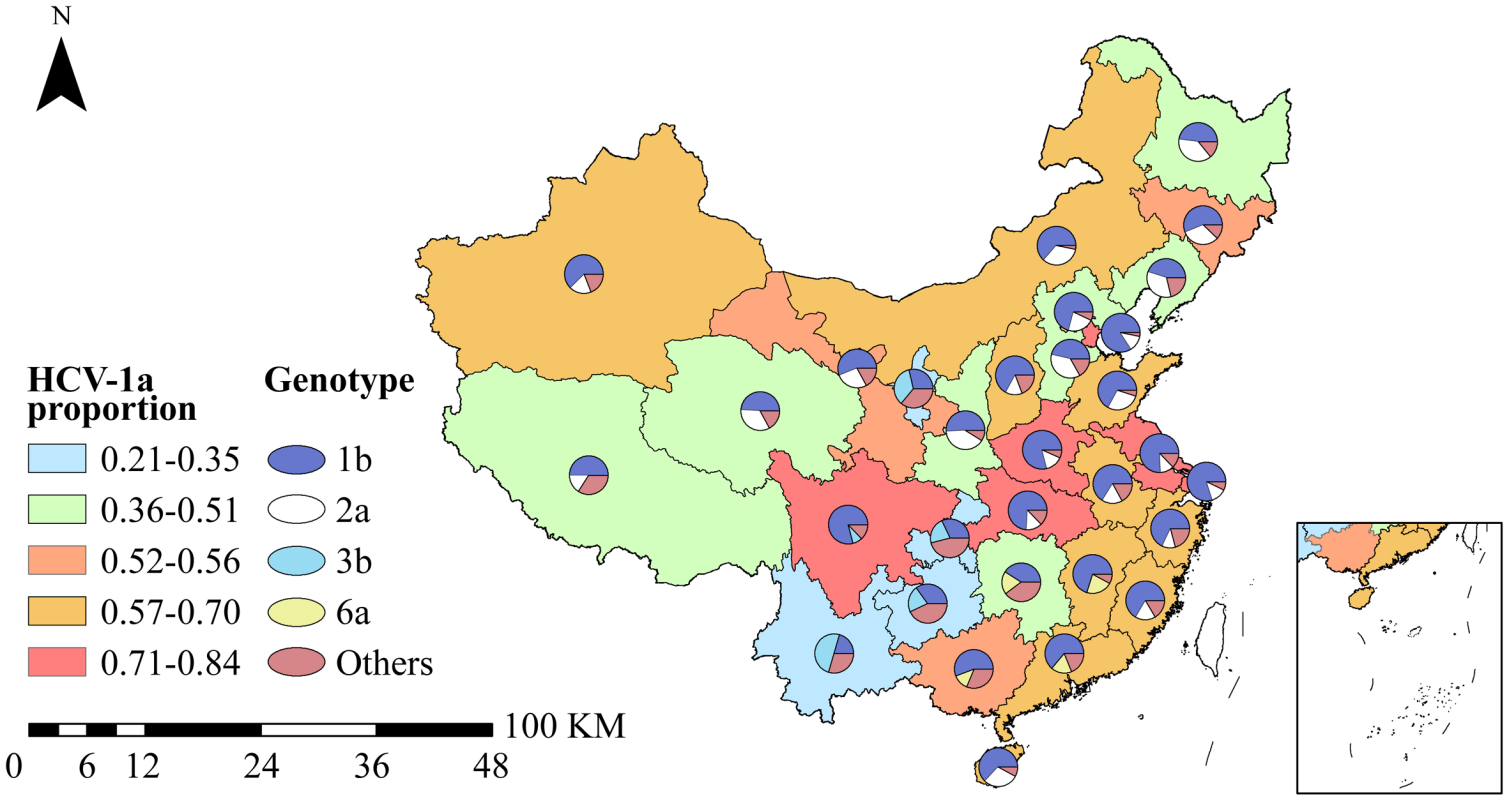
Distribution of HCV genotypes in China. The color of map background adopts a square legend, indicating proportion genotype 1a of HCV to all genotype in each province. Pie charts represent the proportion of five genotypes of HCV in each province.

Hepatitis C virus is classified into seven genotypes (HCV 1–7), including 67 established subtypes and 20 provisional subtypes (Messina et al., 2015). Genotype 1 is the most prevalent globally (46.2%) followed by genotype 3 (30.1%). Most Asian countries also display a predominance of HCV-1 and HCV-3 (McOmish et al., 1994). HCV subtypes 1–6 have been identified in China, with 1b, i.e., 62.78% (95% CI: 59.54%–66.02%) and 2a, i.e., 17.39% (95% CI: 15.67%–19.11%) being the two main subtypes (Zhang et al., 2017a). The distribution of genotypes varied greatly across the country (Figure 4). The provinces with the highest proportion of 1a subtypes were Tianjin (84%), Shanghai (80%), Sichuan (79%), Henan (79%), and Hubei (74%).

## Forms of Hepatitis C

**Figure 5:**
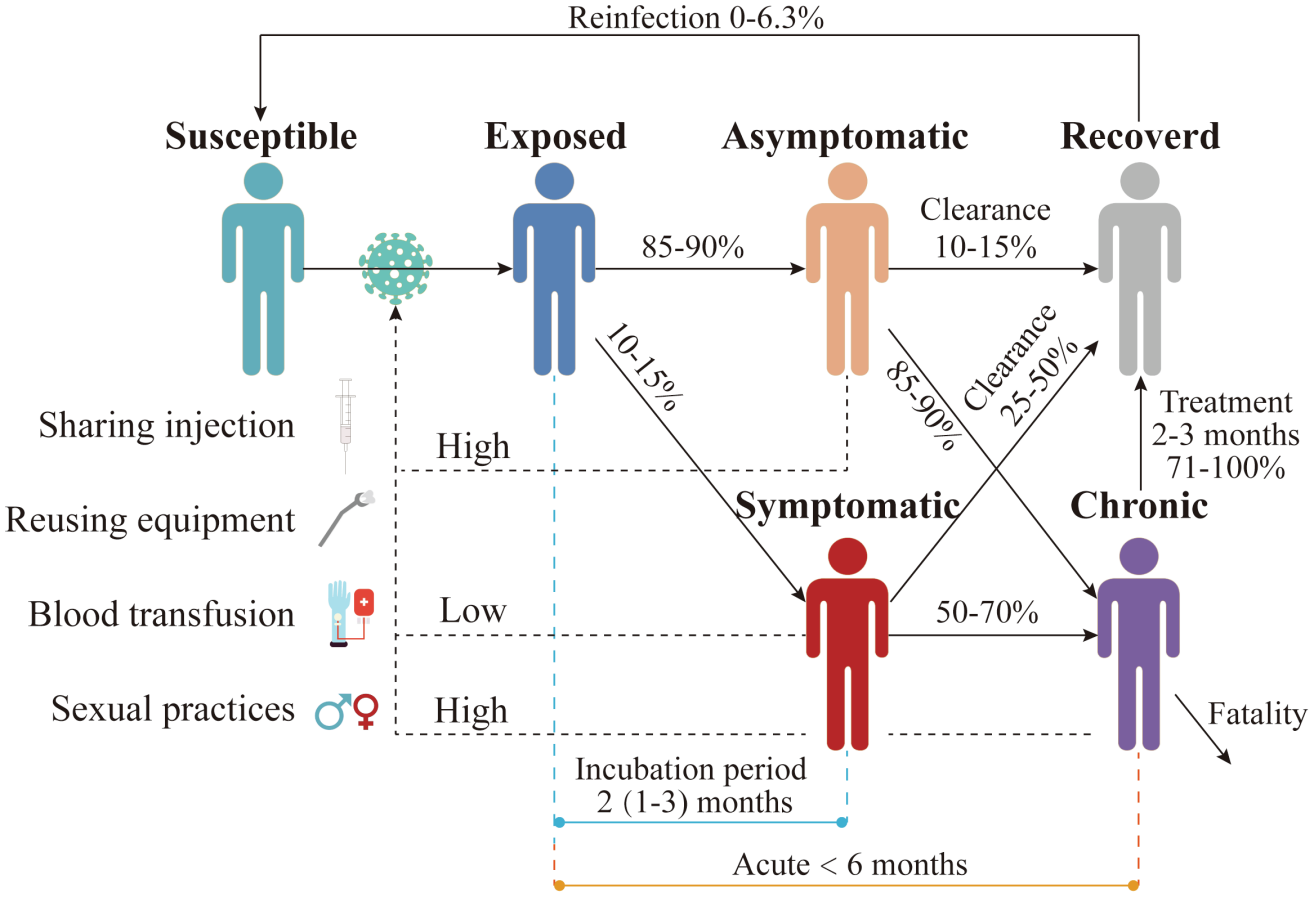
Natural history of HCV infection in humans. People with different colors indicate the infection status of HCV. The solid lines represent the transition of each status, and the numbers above them represent the transition ratio or time. The dotted lines indicate the main transmission routes. “High” means the main transmission route, and “low” means the secondary route.

Hepatitis C is divided into two forms, i.e., acute and chronic hepatitis C. However, asymptomatic people remain the main source of contamination whatever the form might be. Therefore, it is essential to analyze the transmission of HCV caused by asymptomatic carriers (Figure 5). As already highlighted, HCV is mainly transmitted through used syringes and needles, reuse of medical equipment, blood transfusions, and unprotected sexual contact (World Health Organization, 2020). However, mother-to-child transmission is rare. Acute hepatitis C is defined as the first 6 months after initial infection (Hajarizadeh et al., 2013), when HCV is detectable in peripheral blood in susceptible individuals after 1–3 weeks of exposure (Farci et al., 1991). The viral titer will reach a peak after 1–2.5 months (Liu et al., 2012). Viremia is also high at this stage. About 10%–15% of infected individuals become symptomatic after an incubation period of 2 months (range: 1–3 months). Symptoms last for 2–12 weeks and are usually mild and mainly non-specific such as drowsiness and muscle pain, but jaundice may also occur (Hoofnagle, 1997; Maheshwari et al., 2008). Symptomatic acute patients are easily detected, but the majority (85%–90%) of infections is asymptomatic (Ozaras and Tahan, 2009). Therefore, misdiagnosis is a frequent occurrence. Approximately 10%–15% of asymptomatic patients, as well as 25%–50% of symptomatic patients, will experience spontaneous clearance of HCV within the first 3 months (Maheshwari et al., 2008; Ozaras and Tahan, 2009). Spontaneous clearance of hepatitis C virus is more likely to occur in younger age groups and children (Corey et al., 2006; Micallef et al., 2006). Spontaneous clearance of hepatitis C virus infection is approximately twice as likely in women as in men (Kong et al., 2014; Xiong et al., 2017).

Hepatitis C chronicity is defined as the persistence of the hepatitis C virus in the bloodstream for more than 6 months. About 55%–85% of acute infections will evolve into a chronic stage (Chinese Society of Hepatology, Chinese Medical Association, and Chinese Society of Infectious Diseases, Chinese Medical Association, 2020; World Health Organization, 2020). Both asymptomatic (85%–90%) and symptomatic (50%–70% of infected individuals) may develop chronic hepatitis C after 4 months (range: 3–5 months) (Maheshwari et al., 2008; Ozaras and Tahan, 2009). Most chronic hepatitis C patients are also asymptomatic, making this population significant for the impact of hepatitis C transmission. A very small percentage of chronic hepatitis C patients are self-clearing, but recovery rates of 71%–100% can be achieved after 2–3 months of treatment (Grebely et al., 2011). However, patients who develop severe lesions may die from the disease. Although HCV infection can be spontaneously cleared and recovered with treatment, the risk of secondary infection remains, with the likelihood of secondary infection varying from 0 to 6% (Martinello et al., 2017). In addition, patients coinfected with HIV and HCV are at greater risk of secondary infection (3%–15%; Westbrook and Dusheiko, 2014; Martinello et al., 2017).

The parameters of the natural history of HCV in China are also consistent with those estimated by other countries. In China, approximately 40 million people are infected with HCV (Li et al., 2012), and it is estimated that 50%–85% of all individuals infected with HCV develop chronic hepatitis. Another study indicated that the proportion of acute patients who are transformed into chronic patients is about 82.62% (Jia et al., 2019). In Hebei Province of China, a 9-year follow-up of hepatitis C virus infection study found that 12 (8.4%) of 142 cases were negative for both HCV RNA and anti-HCV (Fan et al., 2004). Another study showed that 92 in 416 cases (22.44%) would occur spontaneous clearance after at least 6 months (Kong et al., 2014). Meanwhile, the spontaneous clearance of HCV in 100 HCV-infected Chinese blood donors was 24.0% in China (Liu et al., 2013). A study reported that the mean incubation period of 99 HCV-infected patients was close to 31–45 days in a county of China. The Chinese guidelines for the prevention and treatment of hepatitis C summarized the rate of sustained virologic response (SVR) in hepatitis C patients after 12 weeks of DAAs treatment and found that most DAAs were highly effective (more than 85%; Chinese Medical Association and Chinese Society of Infectious Diseases, 2019). Furthermore, a study showed that an intention-to-treat analysis of a real-life observational study revealed more than 90% of SVR rates of 192 patients in China (Zeng et al., 2017).

## Prevention and Control Strategies in China

There is no effective vaccine to prevent HCV infection. According to the HCV infection prevention and control guidelines in 2019, the main prevention and control measures for hepatitis C include screening and management, strict screening of blood donors, prevention of medical and broken skin-mucous membrane transmission, prevention of sexual transmission, prevention of mother-to-child transmission, and active treatment and management of infected individuals (Chinese Society of Hepatology, Chinese Medical Association, and Chinese Society of Infectious Diseases, Chinese Medical Association, 2020).

### HCV Infection Prevention and Control Policy in China

China has adopted some active strategies to strengthen HCV infection prevention and control. HCV infection elimination has been included in the “Healthy China 2030” plan (The State Council of the People's Republic of China, 2016). China has established HCV Infection Elimination Alliance in 2017, and the established pilots to conduct free screening for HCV in high-incidence counties (People 's Daily Online, 2017). The government also issued guidelines for HCV infection prevention and treatment in 2019. This series of policies emphasizes the determination and efforts to eliminate HCV infection in China.

### Early Screening for HCV

Early screening for HCV infection is very important and is currently focused on high-risk groups, such as drug users, blood transfusion recipients, and renal dialysis patients (Alter and Margolis, 1998). The diagnosis rate of HCV in China is low. In 2016, only 18% of HCV-infected people were diagnosed, which is significantly lower than the WHO target of 72% diagnosis rate (Virtual Community of Hepatitis C, 2017). One study advocates that the entire general population should be screened for HCV as screening is still lacking even in high-risk populations (Mak et al., 2018). In addition, the presence of a large proportion of asymptomatic infected individuals further increases the difficulty of efficiently screening for HCV (Ozaras and Tahan, 2009).

### Positive Treatment and Management of HCV Infection

Although early screening can greatly reduce the HCV transmission, the prevention and control of HCV should still be based on active treatment. Most studies used SVR to assess the effectiveness of the treatment (Miriam and Harold, 1998). DAAs are considered ideal treatment for people with chronic HCV infection (SVR > 90%; Virtual Community of Hepatitis C, 2017). The WHO recommended that all patients over 18 with chronic hepatitis should be treated with DAAs (World Health Organization, 2016). DAAs therapy cures most people with HCV infection within a short period of time (usually 12–24 weeks; Mak et al., 2018). According to WHO, about 5 million people were treated with DAAs as of 2017, but this was still far from the global goal of achieving 80% treatment by 2030 (Ozaras and Tahan, 2009).

The genotypes of hepatitis C in China are mainly type 1 and type 3. According to the new Chinese guidelines of hepatitis C, the SVR12 rates for genotypes 1a, 1b, 2, 3a, 3b, and 6 were 100, 100, 100, 95, 76, and 99%, respectively, after 12 weeks of treatment with sofosbuvir/velpatasvir (Chinese Medical Association and Chinese Society of Infectious Diseases, 2019). There is no vaccine to prevent HCV, but combination therapy with DAAs can cure more than 95% of chronic HCV (Chinese Hospital Infection Control Branch of Preventive Medicine, Chinese Society Branch of the Medical Association, Chinese Society Prevention and Control Branch of Preventive Medicine, 2021). In general, DDAs are very effective against hepatitis C, with most drugs achieving and SVR rate of more than 80% after 12 weeks of treatment. Most DAAs are already approved in China. China’s HCV elimination plan in September 2021 also identifies a cure rate of more than 95% of HCV patients within 10 years (China Liver Health, 2021).

### The Management Systems of Hepatitis C Prevention and Treatment in China

To widely promote the screening and management of HCV-infected patients, several platforms have been established in China using information technology, such as the HCV community-self-management system for patients with liver disease, and the HCV Screening and Management Information Platform of Shengjing Hospital of China Medical University (Virtual Community of Hepatitis C, 2016; Wang et al., 2018). The HCV Screening and Management Information Platform integrates screening, alerting, reporting, and management intervention functions. These platforms combined with hospital information systems, help achieve regulatory tracking of HCV-infected patients and improve management efficiency.

### Other Measures for HCV Infection Prevention and Treatment

Other prevention and control measures have been implemented to further reduce the incidence of hepatitis C, such as the popularization of condoms, the strict control of nosocomial infections, the enhanced management and health education for HCV-infected patients. It is vital to increase public awareness of the prevention and control of hepatitis C (Chinese Society of Hepatology, Chinese Medical Association, and Chinese Society of Infectious Diseases, Chinese Medical Association, 2020).

## Suggestions for the Improvement of the Prevention and Control of HCV in China

China still has a long way to go to achieve the WHO goal of hepatitis C elimination by 2030. China has proposed a work program for the elimination of hepatitis C from 2021 to 2030, with seven key tasks, including strengthening HCV awareness and education, providing comprehensive interventions for key populations, enhancing standardized treatment and improving treatment rates, strengthening medicine supply, establishing and improving information management systems, and increasing detection capacity and implementing medical insurance policies (Health Commission of Hebei Province, 2021). To further reduce the burden of hepatitis C in China, we make the following recommendations (Table 1).

1. Screening for HCV should be conducted in the general population, to increase the diagnosis rate and thus improve the effective control of HCV. For example, the American CDC recommended a one-time HCV screening for all individuals born between 1945 and 1965 (AASLD/IDSA HCV Guidance Panel et al., 2015). Similar action should be implemented. In addition to increasing screen rates based on risk factors, prenatal screening of pregnant women also needs to be reinforced (World Health Organization, 2017b).
2. Hepatitis C virus epidemic is usually exacerbated by the transmission of the virus among those who are vulnerable and at high risk, such as drug users, sex workers, the MSMs, and minorities. The optimal, cost-effective intervention comprises screening of vulnerable populations, ensuring that all individuals newly diagnosed with HCV receive DAA (Heffernan et al., 2021). We recommended strengthening the screening in vulnerable and high-risk populations. Screening of people at high risk for HCV could more effectively identify people with HCV infection and thus provide an entry point for them to access care, treatment, and support. It might be useful to develop mathematical models and AI systems to prioritize more vulnerable and high-risk populations (gender, age class, occupation, comorbidities, genotype, etc.). Further studies could provide reasonable screening protocols, such as the comprehensive screening program recommended in the United States for high-risk age groups.
3. The long-term regular management and follow-up of HCV-infected patients also need to be further strengthened. Health managers may consider improving national HCV surveillance management system to guarantee the attendance and recovery rate of HCV-infected patients.
4. Although efficient therapies for HCV already exist, they are expensive in certain cases (Li et al., 2019). Treatment costs need to be reduced and DAAs should be considered for inclusion in health insurance.
5. Some scholars have noted that integrating HCV healthcare and surveillance systems, strengthening primary care level, and coordinating government initiatives are essential in providing effective HCV preventive measures and treatments (Chow et al., 2014; Sun et al., 2021). Disparities in healthcare resources across the provinces increased following the economic reform, and the socioeconomic status is associated with healthcare resources (Bakkeli, 2021). The eastern regions with better economic status and more healthcare investment have high-quality healthcare resources (Guo et al., 2021). Residents from the affluent eastern regions are more likely to use preventive care than those from the central and northeastern regions (Huang et al., 2016). Rural residents have significantly less access to healthcare services than urbanites (Zhu et al., 2017; Zhang et al., 2017b). Thus, regional-specific strategies should be proposed and conducted to prevent HCV infections and manage HCV patients, based on local HCV prevalence, government healthcare funding, and disparities in healthcare access.
6. To achieve hepatitis C elimination, hepatitis C prevention and control strategies have been proposed and implemented in most regions of China. In accordance with China’s HCV prevention and control strategy, some provinces with high incidence rates had further strengthened prevention and control. For example, Guangdong has increased its emphasis on mobile big data to implementation of education and monitoring. Henan emphasized the role of health education and opinion leaders. Several provinces have initiated sentinel surveillance for HIV and hepatitis C. As of November 2020, Qinghai Province has established 21 national sentinel sites, covering basically all provinces for all types of high-risk populations (The People's Republic of China, 2020). Zhejiang Province decided to launch basic medical insurance for hepatitis C (antiviral treatment) outpatient medical expenses to be paid by disease type in 2019 [Notice on Carrying out Basic Medical Insurance Hepatitis C (Antiviral Treatment) Outpatient Zhejiang Provincial Medical Security Bureau, 2019]. Tianjin launched a capitation payment scheme for outpatient medical institutions for hepatitis C and a per-case payment scheme for outpatient medical institutions (The People's Republic of China, 2018).

**Table 1:** Recommendations to further reduce the burden of hepatitis C in China.

Screening and management	Strengthen the screening of hepatitis C in high-risk groups to improve the awareness of prevention and treatment of the general population. Testing of donated blood.
	(1) High-risk populations. We recommended that health agencies emphasized testing in vulnerable populations, such as drug users, sex workers, and minorities. (2) Increase testing rates in the general population.
	Closed-loop management of screen-positive patients through a multi-sectoral collaborative screening and referral pathway for HCV infection.
Treatment	Three DAAs had been covered in national health insurance, including glatavir/elbasvir (genotype 1/4), sofosbuvir/velpatasvir (genotypes 1–6), and sofosbuvir/ledipasvir (genotype 1/4/5/6).
Healthcare setting	Hand hygiene: including surgical hand preparation and hand.
	Safe cleaning of equipment.
	Safe handling and disposal of sharps and waste.
	Improved access to safe blood.
	Reducing the exposure of the newborn to maternal blood to prevent mother-to-child transmission.
Health education and training of health personnel	Prevention of sexual contact transmission. (1) Managed MSM and people with multiple partners. (2) Universal condom use. (3) Provided proper sex education to adolescents.
	Counseling patient education on prevention and control of HCV to improve diagnosis and treatment rates.
	Regular training on the topic of occupational exposure to HCV infection.

## Conclusion

This review elaborated the feasibility of hepatitis C elimination in China from an epidemiological, natural history, and intervention perspectives. From 2005 to 2012, the incidence of HCV in China showed an increasing trend, with a stable trend after 2012. The disease burden of HCV is usually heavy among those vulnerable and high-risk populations such as drug users, sex workers, the MSMs, minorities, etc. By interpreting the natural history of hepatitis C, we suggest that the focus should be on transmission from asymptomatic populations. Improving the diagnosis rates, treatment, management, and health education in high-risk populations and asymptomatic individuals is essential to slow or even curb the HCV epidemic. The country should pay more attention to establishing sentinel sites for HCV to observe the epidemic and evaluate interventions. The spatial distribution of hepatitis C incident rates indicates regional disparities, with the higher incidence rates concentrated on the northwestern part of China. Several factors such as health care, socio-economy, and customary practices, may contribute to differences in hepatitis C prevalence, screening, and treatment. The eastern regions with better economy may spend more on health care. Therefore, regional-specific surveillance and management system should be established to better prevent and control the transmit of HCV.

## Author Contributions

TC and RF conceptualized and designed the study. ZZ and MC prepared the manuscript and figures. ZZ, MC, YG, SY, and GA contributed to literature search. TC, RF, YG, SY, and GA contributed to the revision and reviewed the final manuscript. All authors contributed to the article and approved the submitted version.

## Funding

This study was supported by the Bill and Melinda Gates Foundation (grant no. INV-005834 to TC).

## Conflict of Interest

The authors declare that the research was conducted in the absence of any commercial or financial relationships that could be construed as a potential conflict of interest.

## Publisher’s Note

All claims expressed in this article are solely those of the authors and do not necessarily represent those of their affiliated organizations, or those of the publisher, the editors and the reviewers. Any product that may be evaluated in this article, or claim that may be made by its manufacturer, is not guaranteed or endorsed by the publisher.
